# Economic valuation of informal care in cerebrovascular accident survivors in Spain

**DOI:** 10.1186/1472-6963-13-508

**Published:** 2013-12-05

**Authors:** Juan Oliva-Moreno, Isaac Aranda-Reneo, Cristina Vilaplana-Prieto, Almudena González-Domínguez, Álvaro Hidalgo-Vega

**Affiliations:** 1 Department of Economic and Financial Analysis, Faculty of Legal and Social Sciences, University of Castilla-La Mancha, Toledo, Spain; 2 Red de Investigación en Servicios de Salud en Enfermedades Crónicas (REDISSEC), Toledo, Spain; 3 Department of Economic and Financial Analysis, Facultad de Ciencias Jurídicas y Sociales, Universidad de Castilla-La Mancha, Cobertizo San Pedro, Mártir, Toledo s/n 45071, Spain; 4 University of Murcia, Murcia, Spain; 5 Biostatistics Department, Max Weber Institute, Majadahoda, (Madrid), Spain

## Abstract

**Background:**

Cerebrovascular diseases are the second leading cause of death worldwide and one of the health conditions which demand the highest level of social services. The aim of this study was to estimate the social cost of non-professional (informal) care provided to survivors of cerebrovascular accidents (CVA) with some type of disability in Spain.

**Methods:**

We obtained data from the 2008 Survey on Disability, Independent Living and Dependency (EDAD-08) on the main characteristics of individuals who provide informal care to survivors of CVAs in Spain. We estimated the cost of substituting informal care in favor of formal care provided by professional caregivers (proxy good method) and performed a statistical analysis of the relationship between degree of dependency and number of care hours provided using ordinary least squares regression.

**Results:**

The number of disabled people diagnosed with CVA totaled 1,975 (329,544 people when extrapolating to the national population using the elevation factor provided by EDAD-08). Of these, 1,221 individuals (192,611 people extrapolated to the national population) received at least one hour of informal care per week. The estimated hours of informal care provided in 2008 amounted to 852 million. The economic valuation of the time of informal care ranges from 6.53 billion euros (at 7.67 euros/hour) to 10.83 billion euros (when calculating each hour of care at 12.71 euros). The results of our statistical analysis highlight the importance of degree of dependency in explaining differences in the number of hours of informal care provided.

**Conclusions:**

The results of our study reveal the high social cost of cerebrovascular accidents in Spain. In addition, evidence is presented of a correlation between higher degree of dependency in CVA survivors and greater number of hours of care received. An integral approach to care for CVA survivors requires that the caregivers’ role and needs be taken into account.

## Background

Cerebrovascular diseases (CVD) are the second leading cause of death after ischemic heart disease (IHD), and the third leading cause of disability-adjusted life years in high-income countries after IHD and lower respiratory infections. The World Health Organization estimated that in 2010 CVD caused 5.87 million deaths across the globe (11% of all causes of death) and 102 million disability-adjusted life years [1,2].

In Spain, CVA is the leading cause of death among women and the third among men [3]. In addition to deaths, the effects of the disease on survivors and consequentially the loss in quality of life must be taken into consideration when assessing its impact [4,5]. People who survive a CVA usually suffer negative long-term effects which, in many cases, reduce the person’s autonomy and lead to functional dependency [6].

Reviews of CVA cost studies report that there are fewer economic analyses of the costs of CVA than there are for other diseases [7-9]. However, the available studies stress that CVA is an acute and highly costly condition which accounts for a significant portion of overall healthcare spending. Evers et al. estimated that 3-4% of health spending in high-income countries is spent on CVA, mainly reflecting the direct cost of hospitalization during the first year of survival [9]. However, studies that address the cost of CVA from a broader (social) perspective indicate that non-medical costs (formal and informal care and productivity losses) may far outstrip medical costs [10-13].

As far as we know, only one study published to date has applied a cost-of-illness design using a national sample to CVD in Europe [14]. This study revealed that non-healthcare costs accounted for 38.9% of the overall costs of the disease, with informal care being the most substantial item among non-healthcare costs (54.4% of this item). Studies which calculate such global estimates are uncommon in the literature, since they require very rich sources of data. In Spain, research has been published describing informal care in patients who have had a CVA, although these studies were either performed on small samples of patients or used mathematical models [4,15-17], and only one such study [15] analyzed the influence of the degree of dependence on care hours received. Informal care is a societally relevant resource, since any absence of informal caregivers requires the care time needed by the patient to be substituted by professional social services (formal care). Obtaining data on informal care rendered would reveal the reduced societal burden of CVA avoidance or, in cases of CVA occurrence, the data would help prevent patients from progressing to a state of great dependence.

The main purpose of this study was to assess the social cost of informal care associated with the loss of autonomy (dependency) among CVA survivors in Spain and to study the association between the degree of dependency and the total number of hours of care received by these individuals.

## Methods

The Survey on Disability, Independent Living and Dependency 2008 (EDAD-08) was designed by adapting the 1999 edition of the Survey on Disabilities, Impairments and State of Health to the current social and demographic situation and the new International Classification of Functioning, Disability and Health [18]. The main improvement of EDAD-08 over the 1999 study is its adaptation to the new International Classification of Functioning, Disability and Health (ICF) [19]. Previously, interviewees were asked directly if they had a disability. In the EDAD-08 study, however, questions examined the limitations in activity experienced by the subject, as proposed by the ICF. Interview questions like those in the EDAD-08 study make it possible to identify the limitations and disabilities from a wider and more objective point of view.

The households section of the EDAD-08 study consists of various questionnaires. These include the Household Survey (first phase), followed by two individual surveys, the Survey on Disabilities for persons over 6 years of age and the Survey on Limitations for children up to five years on the one hand, and a questionnaire for the primary caregivers on the other (second phase). The data were collected between November, 2007 and February, 2008 and all questionnaires and results are available on Spanish National Statistics Institute website [18]. In order to fulfill the objective of providing national, regional, and provincial estimates with a certain degree of reliability, the survey sample was comprised of 96,075 households and a stratified two-stage sampling was used (for details, see reference [20]). Of the total sample, 22,795 persons with disabilities were identified and interviewed in depth. Personal interviews were used to collect individual information; in exceptional cases, these face-to-face interviews were complemented with telephone interviews. Among the variables included in the EDAD-08 survey were the personal characteristics of the persons with disabilities (including whether or not they received personal care and, if so, the length of time of the care received), characteristics of the caretaker persons (when identified), and which activities were provided under informal care.

The number of disabled people diagnosed with CVA amounted to 1,975 individuals (329,544 people extrapolated to the national population). Of these, 1,221 (192,611 people extrapolated to the national population) received at least one hour of informal care per week.

In this study, *informal care* was defined as the attention provided to an individual with limitations in autonomy when conducting one or more of his/her daily activities, this attention being provided by persons who are not professional social workers. The key attribute of these care persons is that the determining factor causing the informal caregiver to accept the role is a family or social bond between him/her and the person with limited autonomy. Receiving compensation from another family member for these services does not disqualify the service as non-professional in nature.

In order to classify individuals who have limitations in their autonomy by degree of severity, a correspondence was assigned between the questions contained in the EDAD-08 survey and the Official Dependency Scale of Spain [21].

### Valuation methods

An assessment of informal care requires that a distinction be made between the cost of care provided by informal caregivers and other care costs, such as home adaptations or the acquisition of medical equipment (e.g., wheelchairs, walkers, adjustable beds, special telephones, etc.), all of which are common elements in disabling diseases. The cost estimates in this study were performed using data relating only to the time spent by caregivers providing care. There are different methods for assigning value to time [22-24]. Given that the EDAD-08 survey provides data to calculate the hours of care provided, we have applied the proxy good method to use these data in our research [24]. The proxy good method or market cost method values time spent on informal care based on the labor market prices of a similar market substitute. Services provided by informal caregivers were assigned a value based on what it would cost to have a professional caregiver provide the services. Doing so allowed us to determine how much it would cost to replace the informal caregivers with professionals.

Using 2008 as our base year, we designed three scenarios for estimating replacement or substitution costs. In the *first scenario*, the hours of care were valued at the average official wage for social services home care in the 3 autonomous regions of Spain with the lowest hourly wage [25]. The value used was 7.67 euros per hour of care. In the *second scenario*, we used the average hourly wage reported by all of the autonomous regions. The average hour of care was estimated at 12.71 euros. In the *third scenario*, the same government source was used to determine the hourly wage for care in each autonomous region. These regional wages ranged from 6.2 to 22.8 euros.

The number of hours of informal care was assessed by carefully applying various criteria to data contained in the EDAD-08 survey. First, the disabled person—or the person providing information on the household—was asked if the person with the disability received assistance or social care because of his/her disability and the average number of hours per day he/she received help from others, not including the care and services provided at day centers or other services provided by professionals. This question was used to exclude cases in which care was mainly provided by domestic workers, hired healthcare professionals, state-run social services, social services provided by non-governmental entities (NGOs, associations) or for-profit companies. Some survey respondents indicated that the disabled person received social care, however they did not say who provided the care or how many hours of care were given (14% of total CVA survivors who stated they needed personal attention did not indicate the hours of caregiving needed). These cases were not included in our estimate of the hours of informal care. Thus, the estimate was calculated based on the hours of care provided by family, friends, and neighbors (daughters, sons, mother, father, spouse or partner, sisters, brothers, grandmothers, grandfathers, granddaughters, grandsons, other relatives, and friends or neighbors). Lastly, we censored at 16 the maximum number of daily hours per caregiver; that is, we assumed a daily period without care of eight hours per informal caregiver. Therefore, cases where the number of hours of care per day exceeded 16 hours (17–24) were considered as 16-hour days. We also estimated the hours of informal care without applying any censoring—; that is, we did not set a maximum of 16 hours and used the hours of informal care as reported by the people who answered this question. In Spain, the social services home care are made up of a set of initiatives that are performed in the home of the dependent person in order to cater for his/her everyday needs. It includes: (a) services related to attending to domestic or home needs (cleaning, ironing, washing, cooking and others); (and b) services related to personal care, in performing the activities of daily living (bathing, dressing, helping to walk, etc.).

### Statistical analysis

We carried out a statistical analysis to estimate the marginal effect that a higher degree of dependency had on the number of hours of informal care provided to a CVA survivor who had a disability (but not one that would render the person dependent according to the Spanish Official Scale in effect in 2008 [21]). This analysis estimated the number of additional hours of informal care that would be required if his/her dependency status changed to moderately, severely or greatly dependent. For this purpose, we designed several models using different procedures which are explained below. We created two types of estimation models where the variable under study (number of hours of informal care provided by the primary caregiver in a week) was estimated with and without a 16-hour per-day limit.

A multivariate analysis using the number of hours of informal care provided by a primary caregiver per week (not limited to a maximum of 16 hours a day) as the dependent variable was performed using ordinary least squares regression to obtain the Eicker-White heteroscedasticity robust covariance matrix estimate [26]. In Model 1, the control variables were age, sex, and education of the persons receiving care, in addition to the degree of dependency. In Model 1a, a limit of 16 hours per day was applied as a maximum number of informal-care hours. The explanatory variables in this model were the same as in Model 1. In Model 2 (the 16-hour daily limit was applied in Model 2a) the control variables were age, sex, and education of the persons receiving care; degree of dependency; and size of the town of residence. In Model 3 (the 16 hour a day limit was applied in Model 3a), the dependent variables were the same as in Models 2 and 2b, with the addition of the variable of a live-in caregiver. In Model 4 (the 16 hour a day limit was applied in Model 4a), the control variable “region of residence” was added. In Model 5 (the 16 hour a day limit was applied in Model 5a), information was added on whether the person who received informal care also received formal care.

The most complex models can be expressed by the following Equation 1:

$$\begin{array}{ll} H_{i}=\beta_{0} & +\beta_{1}*A_{i}+\beta_{2}*S_{i}+\beta_{3}*E_{i}+\beta_{4}*D_{i}\\ & +\beta_{5}*T_{i}+\beta_{6}*C_{i}+\beta_{7}*R_{i}+\beta_{8}*F_{i}+\epsilon_{i} \end{array}$$

where:

H_i_ = hours of informal care per week (primary caregiver); A_i_ = age of care recipient; S_i_ = sex of care recipient; E_i_ = education of care recipient; D_i_ = degree of dependence of care recipient; T_i_ = size of city or town of residence; C_i_ = live-in caregiver; R_i_ = region of residence; F_i_ = formal care provided to care recipient.

## Results

The number of disabled people diagnosed with CVA amounted to 1,975 individuals. Of these, 1,318 (66%) reported receiving informal care, but only 1,221 (61%) stated that at least one hour of informal care was provided during the week. The results provided below are an extrapolation from this sample to the entire population according to the elevation factor provided by the EDAD-08 survey. Under this extrapolation, the EDAD-08 survey identified 329,500 people in Spain who had survived a CVA and had some type of disability. Of these, 208,865 declared to receive informal care and 192,611 declared to receive at least one hour of care weekly. The main characteristics of care recipients and caregivers appear in Table 1.

**Table 1:** Characteristics of informal caregivers and their care recipients

	**Care recipient**	**Caregivers**
	**Mean (±SD) or %**	**Mean (±SD) or %**
**Age**	73.68 (±16.24)	57.51 (±14.14)
**Sex**		
Male	45.03%	19.34%
Female	54.97%	80.66%
**Marital status**		
Single	48.51%	71.96%
Married	10.80%	16.65%
Widow(er)	38.31%	5.11%
Divorced	2.38%	6.28%
**Education**		
Illiterate	16.69%	3.77%
Incomplete primary studies	40.60%	23.13%
Primary school	32.14%	47.76%
Lower vocational	5.57%	12.97%
Medium vocational	1.69%	2.62%
University	3.30%	9.75%
**Occupation**		
Paid job	1.01%	21.04%
Unemployed	0.67%	8.32%
Disability pension/retired	70.17%	26.81%
Other pension	14.39%	5.19%
Permanent disability	4.10%	0.79%
Housework	0.27%	0.55%
Other	5.90%	35.57%

Survey respondents reported impairments or disabilities in various dimensions: hearing (26.4%), sight (31.2%), interpersonal relations (30.1%), learning (33.9%), communication (39.2%), self-care (74.2%), housework (74.9%), and mobility (85.6%). According to our estimates, in 2008, 23.1% of CVA survivors would not be considered dependent under the Official Dependency Index criteria [21], 23.7% suffered a moderate degree of dependency, 20.6% were classified as severely dependent and a 32.6% as greatly dependent (Table 2).

**Table 2:** Official dependency index criteria

**Degree of dependence**	**Care recipient, at least one hour per week (n = 1,221/192,611)**	**All CVA survivors (n = 1,975/329,544)**
Not dependent	23.1%	43.6%
Moderately dependent	23.7%	18.3%
Severely dependent	20.6%	15.6%
Greatly dependent	32.6%	22.6%

Note: results are an extrapolation to the entire population according to the elevation factor provided by the EDAD-08 survey.

It is important to note that primary informal caregivers who provided at least one hour of caregiving during the week (n = 192,611) represent a considerable number of hours of care; 96.1% stated that they provided care 6 or 7 days a week for an average of 11.3 hours a day. Consequently, many caregivers bear heavy burdens: 42.0% believed that their health had suffered; many stated that they felt tired (55.4%) or depressed (34.3%); three out of ten caregivers were not able to work outside the home due to the demands of caregiving; 17.1% reported having had to stop working; 12.1% thought their careers had been negatively affected; and one in four caregivers thought that they had experienced financial difficulties owing to the care they gave. Furthermore, seven out of ten caregivers thought that their free time had been reduced significantly and, more specifically, 53.0% stated they could not go on holiday, and 39.7% of caregivers had no time to take care of themselves.

The 2008 estimated hours of informal care in Spain amounted to 852 million. The greatest proportion of these hours was provided by primary caregivers (731.8 million hours of informal care, or nearly 86% of the total). The rest of the time was provided by other informal caregivers. Applying our cost-assessment methodology, the economic valuation ranges between €6.53 billion (scenario 1) and €10.83 (scenario 2). The economic valuation estimated in scenario 3 (€10.37 billion) closely resembles the figures estimated in scenario 2. Estimates for each of the autonomous regions in Spain are available upon request (Table 3).

**Table 3:** Hours of informal care per week (primary caregiver)*

	**Model 1**	**Model 1a**	**Model 2**	**Model 2a**	**Model 3**	**Model 3a**	**Model 4**	**Model 4a**	**Model 5**	**Model 5a**
Moderately dependent	29.893 (4.718)	22.348 (3.171)	29.460 (4.729)	22.167 (3.170)	26.230 (4.675)	20.128 (3.137)	26.557 (4.624)	20.466 (3.091)	26.148 (4.645)	20.189 (3.100)
Severely dependent	53.478 (4.432)	38.665 (2.862)	53.261 (4.434)	38.532 (2.861)	49.231 (4.404)	35.988 (2.849)	48.427 (4.401)	35.659 (2.818)	48.141 (4.430)	35.414 (2.826)
Greatly dependent	59.253 (4.051)	40.499 (2.658)	59.090 (4.051)	40.521 (2.656)	55.222 (4.037)	38.079 (2.648	55.574 (4.017)	38.569 (2.622)	55.382 (4.149)	38.327 (2.692)
N	1265	1265	1265	1265	1265	1265	1265	1265	1265	1265
F	29.5	32.37	21.26	23.60	30.12	30.07	16.66	17.01	15.86	16.21
R^2^	0.1686	0.1996	0.1726	0.2041	0.2143	0.2454	0.2355	0.2653	0.2378	0.2675

*Heteroscedaticity robust covariance matrix estimate (using Ordinary Least Squares) (standard deviation between brackets). Omitted category: Persons with disabilities not classified as dependent according to the Spanish Official Scale. Control variables for each model: See Methods Section.

The statistical analysis underscores the importance of degree of dependency in explaining differences in the number of hours of care provided. In the models where the maximum number of hours per day was limited to 16, our results show that compared to our reference group (informal care provided to persons not classified as dependent according to the Spanish Official Scale), a person who is moderately dependent would receive 20–22 additional hours of care per week from their primary informal caregiver. In the case of severely dependent individuals, the primary informal caregiver would provide 35–38 additional hours of care. Primary informal caregivers would provide an additional 38–40 hours of care per week to recipients classified as greatly dependent.

In the models where the number of hours per day was not limited to a pre-established maximum, the results show that compared to the reference group (informal care provided to persons not classified as dependent), a person who is moderately dependent would receive 26–30 additional hours of care per week from their primary informal caregiver. In the case of individuals who are severely dependent, the primary informal caregiver would provide 48–53 additional hours of care. Primary informal caregivers would provide an additional 53–59 hours of care per week to care recipients classified as greatly dependent.

## Discussion

Our study estimates the total cost of non-professional care provided to persons who have suffered a CVA, using the Survey on Disability, Independent Living and Dependency 2008. The present study is the first to use an official scale to identify the degree of dependency of people with CVA. Although other works have evaluated informal care in CVA taking into account level of dependency, methodological differences between the studies, including the use of different dependency scales, prevents the comparison of their respective results [15,27,28]. On the other hand, other works with a robust methodology for measuring costs and which have included the cost of informal care, did not control for level of dependency in CVA survivors [14,16,29-32].

Originally, Spain provided low levels of social protection expenditure for long-term care (CLD) compared to other European countries [33]. Thus, the Spanish care model for people with limitations on their autonomy relies to a much greater extent on family care (informal care) than do other European countries, which devote greater resources to professional care [34]. This discrepancy must be considered before extrapolating the findings of this work to other countries, where it is expected that the number of hours of informal care in patients surviving a CVA would be less, with greater involvement of care professionals.

In order to appreciate the relevance of our results, we need to look at other estimates of the cost of total formal care provided for dependent people in Spain. Jiménez-Martín and Vilaplana estimated that the total cost of formal services provided between 2008 and 2009 totaled 6.34 billion euros [35]. According to the Spanish Ministry of Health and Social Policies, the cost of the System for the Promotion of Personal Autonomy and Care for Dependent Persons (*Sistema para la Autonomía y Atención a la Dependencia*, or SAAD) in 2010 amounted to 6.77 billion euros [36]. Although the legislative Act on the Promotion of Personal Autonomy and Care for Dependent Persons has driven the growth of spending on dependent care for communities and local government, this financial support was already earmarked before the passage of the law; dependent-care support in 2003 represented 0.32% of GDP, raising to 0.64% in 2010 [36]. Even with the legislation in force, these figures are paltry when compared to the cost of informal care for CVA survivors cited in this study.

Therefore, a comprehensive policy to provide care to dependent people must take into account the role of and care provided by primary caregivers, recognizing the contributions they make. Other authors have made similar suggestions in this regard [37,38]. Policy makers in both the public and private spheres should take note of the fact that the excess burden borne by caregivers generates a host of problems for these persons’ health, career, leisure time, and family life. Strategies designed to mitigate these problems would bolster informal support networks, thereby helping to improve the wellbeing of caregivers as well care recipients while facilitating efforts to coordinate social, health, and family services.

Some limitations of this paper should be stressed. Firstly, the EDAD-08 survey, while an excellent work that serves as a rich source of information on CVA survivors and their disabilities, contains data that is cross-sectional, not longitudinal. This approach would be a significant impediment for studies on the needs of CVA survivors seeking to compare the care received before and after the CVA or on the impact of comorbidity among significant numbers of CVA survivors. Since a causal relationship between a specific disease and the number of hours of informal care received cannot be established, the estimated hours of care must not be attributed solely to the effects of CVAs. Thus, this study does not refer to the need for informal care caused by CVAs; rather, it assesses the care provided to persons who have suffered CVAs. This distinction is important.

The authors have censored to a maximum of 16 hours per day the informal care provided by a primary caregiver. This limit assumes that the caregiver has at least eight hours for rest, personal care, and other activities. Since in many cases this time may be less than indicated, setting this limit would provide a conservative estimate of the time of informal care. Although it seems fair to suggest that daily care time can be 24 hours, and despite the fact that the time spent on tasks belonging to the category of “joint production” must be considered, the decision of the authors to choose a limit of 16 hours is modifiable. In the economic valuation analysis performed, several thresholds (12, 14, 16, and 18 hours) can be employed as a sensitivity analysis depending on whether the analysts prefer to be more or less conservative in their estimations.

Another point worth noting is that estimates were performed using a proxy good method based on hourly wages. Another option would have been to assign values based on tasks performed by the caregiver. However, this was not possible because while the EDAD-08 survey does contain questions on the various tasks performed by caregivers, it does not ask respondents to specify the amount of time spent on each.

It also would have been worthwhile to explore methodological issues by drawing comparisons of different methods for valuing time (e.g., proxy good method, opportunity cost, and contingent valuation) or different ways of calculating hours of care (e.g., diary method, recall method). In any event, the EDAD-08 survey does not contain data with which to analyze these questions.

Another limitation of the analysis is seen in the extrapolation of results to the national level, using the elevation factors contained in the EDAD-08 survey. As in any study based on a given sample which is then extrapolated to a larger population, the results are subject to a margin of uncertainty.

## Conclusions

The results reveal that despite limiting the maximum number of hours, CVA survivors in Spain received 852 million hours of informal care in 2008. Regardless of the estimated cost per hour of care, informal care comes at a huge cost. We estimated the value of hours of informal care using hourly wages of social services employees as a shadow price, and the monetary value lies in the range of 6.53 to 10.83 billion euros. This sum could be considered the minimum value that informal caregivers contribute to societal well-being with their services.

The results of this study suggest that no strategy, program, or policy designed to promote the health and care of persons with limited autonomy can overlook the important role played by social networks (formed mainly by family members). Their contribution must not be ignored if we are to avoid designing and implementing measures fraught with inefficiency and inequities that could erode the well-being of citizens.

## Competing interests

The authors thank Boehringer Ingelheim Spain for the support provided for this study. Cristina Vilaplana also acknowledges help from project ECO2011-30323-C03-02 and ECO2011-2850 from the Spanish Ministry of Science and Innovation.

## Authors’ contributions

JO conceived of and designed the research, drafted the manuscript and critically revised the manuscript for important intellectual content. IA analyzed and interpreted the data and drafted the manuscript. AG and CV acquired the data and performed statistical analyses. AH handled funding and critically revised the manuscript for important intellectual content. All authors read and approved the final manuscript.

## Pre-publication history

The pre-publication history for this paper can be accessed here:

http://www.biomedcentral.com/1472-6963/13/508/prepub
